# Cognitive performance in patients with Myasthenia Gravis: an association with glucocorticosteroid use and depression

**DOI:** 10.1590/1980-57642020dn14-030013

**Published:** 2020

**Authors:** Annelise Ayres, Pablo Brea Winckler, Laís Alves Jacinto-Scudeiro, Rafaela Soares Rech, Geraldo Pereira Jotz, Maira Rozenfeld Olchik

**Affiliations:** 1 Universidade Federal de Ciências da Saúde de Porto Alegre - Porto Alegre, RS, Brazil.; 2 Hospital de Clínicas de Porto Alegre - Porto Alegre, RS, Brazil.; 3 Universidade Federal do Rio Grande do Sul - Porto Alegre, RS, Brazil.; 4 Faculdade de Odontologia, Universidade Federal do Rio Grande do Sul - Porto Alegre, RS, Brazil.

**Keywords:** Myasthenia Gravis, cognition, cognitive, assessment, glucocorticoids, depression, Miastenia Gravis, cognição, avaliação cognitiva, glucocorticoides, depressão

## Abstract

We investigated the cognitive performance of patients with Myasthenia Gravis (MG) through a cross-sectional study. A battery of cognitive assessments and self-report questionnaires regarding quality of life (QoL), sleep, and depression were applied. The sample consisted of 39 patients diagnosed with MG. The scores showed a predominance of cognitive impairment in the Montreal Cognitive Assessment screening test (MoCA) (66.7%) and in the immediate (59.0%) and recent memory (56.4%) tests. However, after the Poisson regression analysis with robust variance, it was found that patients diagnosed with depression had a prevalence ratio (PR) of 1,887 (CI 1,166‒3,054) for lower MoCA scores, PR=9,533 (CI 1,600‒56,788) for poorer phonemic verbal fluency scores, and PR=12,426 (CI 2,177‒70,931) for the Semantic Verbal Fluency test. Moreover, concerning a decline in short-term memory retention, patients using glucocorticosteroids (GC) and with Beck Depression Inventory scores indicating depression showed PR=11,227 (CI 1,736‒72,604) and PR=0.35 (CI 0.13‒0.904), respectively. No correlation was found between the QoL questionnaire and performance in cognitive tests. We found worse performance in tasks of memory and executive functions in MG patients. These are not associated with the length and severity of the disease. However, a significant prevalence ratio was found for poorer memory performance in patients diagnosed with depression and in those using GC.

## RESUMO.

Investigamos o desempenho cognitivo de pacientes com miastenia gravis (MG) por meio de um estudo transversal. Aplicou-se uma bateria de avaliações cognitivas e questionários de autopercepção sobre qualidade de vida (QV), sono e depressão. A amostra foi composta por 39 pacientes com diagnóstico de MG. Os escores mostraram predominância de comprometimento cognitivo no teste de rastreio Montreal Cognitive Assessment (MoCA) (66,7%) e nas tarefas de memória imediata (59,0%) e recente (56,4%). Entretanto, após a análise de regressão de Poisson com variância robusta, verificou-se que os pacientes diagnosticados com depressão apresentaram uma razão de prevalência (RP)=1.887 (IC 1.166‒3.054) para escores mais baixos no MoCA, RP=9.533 (IC 1.600‒56.788) nos testes de fluência verbal fonêmica e RP=12.426 (IC 2.177‒70.931) no teste de fluência verbal semântica. Além disso, uma associação entre pior desempenho nas tarefas de memória de retenção de curto prazo nos pacientes em uso de glucocorticoides (GC) e com os escores do Beck Depression Inventory indicando depressão, com RP=11.227 (IC 1.736‒72.604) e RP=0.35 (IC 0.13‒0.904), respectivamente. Não foi encontrada correlação entre o questionário de QV e o desempenho em testes cognitivos. Sendo assim, conclui-se que foi observado pior desempenho em tarefas de memória e funções executivas em pacientes com MG. Estes não estão associados ao tempo e à gravidade da doença. No entanto, uma taxa de prevalência significativa foi encontrada para pior desempenho da memória em pacientes diagnosticados com depressão e naqueles em uso de glucocorticoides.

## INTRODUCTION

Myasthenia Gravis (MG) is an autoimmune disease caused by the destruction of nicotinic acetylcholine receptors (nAChRs) at the motor end plates found in striated muscles. Its incidence ranges from 1 to 9 cases per million of the general population. The prevalence rate of MG ranges from 15 to 179 cases per million of the worldwide population. There are no data regarding the national prevalence of the disease in the Brazilian population.^1^ ^,^ ^2^

Although it is a predominantly muscular disease, cognitive impairment in patients with MG has been discussed in the literature. Some studies found cognitive decline in memory,^3^ ^,^ ^4^ ^,^ ^5^ ^,^ ^6^ ^,^ ^7^ ^,^ ^8^ ^,^ ^9^ attention, executive functioning,^8^ ^,^ ^10^ verbal fluency,^8^ ^,^ ^9^ and planning tasks.^6^ ^,^ ^11^ However, there are other studies^12^ ^,^ ^13^ ^,^ ^14^ ^,^ ^15^ that found no difference in the cognitive performance of MG patients when compared to healthy controls.

In a recent systematic review and meta-analysis,^16^ the authors described four main explanations for the cognitive deficits found among many MG patients:

- central pathogenic antibody effect (Abs) against acetylcholine receptors (AChRs);
- for some patients, a lack of certain protective factors such as age, disease severity, and type of treatment;
- mood disturbances;
- the possible effect of nonspecific immunological processes.

Therefore, while some studies have shown a tendency toward cognitive decline in patients with MG, conflicting results have also been published. Thus, considering this lack of clarity in the literature and absence of studies in the Brazilian population, the aim of this study was to investigate the cognitive performance of patients with MG and its association with clinical aspects and quality of life (QoL) in patients with MG.

## METHODS

### Study design

This was a cross-sectional, exploratory study.

### Subjects

Patients were recruited from a neuromuscular diseases outpatient clinic at a hospital in Porto Alegre, Brazil, which is a reference in the treatment of patients diagnosed with MG in the state. Diagnosis of the disease was confirmed by electromyography and/or by the presence of AChR/Musk/Striated Muscle antibodies. Patients were evaluated outside of crisis episodes and on medication, who had been stabilized for at least 6 months. Patients with a history of other primary neurological (*e.g*. transient ischemic attacks, cerebrovascular stroke or epilepsy), psychiatric (*e.g*. major depression), previous serious head injury or any sensory or motor disorder that would preclude psychological testing (such as blindness or deafness). We excluded

The collection period took place between February 2017 and December 2018. All subjects were informed about our research objectives and signed an Informed Consent form. This study was approved by the Central Research Ethics Committee of the hospital, certificate of approval number 120399.

### Procedures

All patients were assessed individually in a room at the hospital’s research center. All patients were evaluated in the afternoon, between 12 and 4pm, and the last dose of pyridostigmine was not registered. The duration of a complete test per patient lasted an average of 40 minutes. This evaluation was always performed by the same previously trained researcher. All instruments and questionnaires used have been translated and validated to suit the Brazilian population.

### Measurements

#### Questionnaires

- Sociodemographic questionnaire: a structured questionnaire used to gather general patient data, such as age, gender, education, length of illness, age of diagnosis, initial symptoms, and marital status;
- MG quality of life scale (MG-QOL 15): a self-report questionnaire specifically designed to assess the quality of life of patients with MG. It has 15 items, with scores ranging from 0 to 60 points. The higher the score, the worse the patients’ perception of quality of life;^17^
- Beck Depression Inventory (BDI): a self-assessment tool used to survey the intensity of depressive symptoms.^18^ To determine each score, the values ​​proposed by Gorenstein and Andrade^19^ were used: less than 10 points - no depression or minimal depression; 10 to 18 points - mild to moderate depression; 19 to 29 points - moderate to severe depression; 30 to 63 points - severe depression;
- Epworth Sleepiness Scale: an 8-item self-report questionnaire which assesses the likelihood of falling asleep in eight situations involving daily activities. The overall score ranges from 0 to 24; scores above 10 suggest excessive daytime sleepiness (EDS).^20^

#### Motor scales

- The Quantitative Myasthenia Gravis Score (QMGS): a clinical scale used as an outcome measure for MG. It consists of 13 items, with a maximum score of 39 points. The higher the score, the more severe the disease;^21^ ^,^ ^22^ ^,^ ^23^
- The Myasthenia Gravis Foundation of America clinical classification (MGFA clinical classification): Patients are organized into 5 classes, ranging from Class I -characterized by any ocular muscle weakness, but no other muscle strength issues - to Class V. This last category defines patients who have undergone intubation, (with or without mechanical ventilation) under circumstances that do not include routine postoperative management. The data obtained using the QMGS was used to classify patients.^21^

#### Cognitive tests

- Mini-Mental State Examination (MMSE): the cutoff points for the Brazilian population for formal education is 28 points for more than 8 years; 5 and 8 years: 26 points; 1 and 4 years: 25 points; and illiterate patients: 20 points;^24^
- Montreal Cognitive Assessment (MoCA): The cutoff point for the Brazilian population is 26 points, with an extra point for individuals with 12 years or less of formal education;^25^
- Semantic Verbal Fluency (SVF): There are specific normative values for Brazilian Portuguese speakers: a score of ≥9 named animals for subjects with up to 8 years of formal education and ≥13 named animals for those with over 9 years of formal education;^26^
- Phonemic verbal fluency (PVF): Normative standard scores for the Brazilian population are classified by age and stratified into different periods of formal education;^27^
- Rey Auditory Verbal Learning Test (RAVLT): Normative standard scores for Brazilian Portuguese speakers are classified by age (20‒59 and over 60 years) and gender (female and male).^28^

#### Medication

The drugs used for the treatment of MG were gathered from the patients’ clinical records and divided into three classes:

- acetylcholinesterase inhibitors (AI): pyridostigmine;
- immunomodulators: Azathioprine, methotrexate, cyclophosphamide, mycophenolate;
- glucocorticosteroids (CG): prednisone.

In addition, patients who were prescribed antidepressants and/or had reports of depression in their medical records were quantified.

### Statistical analysis

Statistical tests were selected according to the distribution of data provided by the Shapiro- Wilk test and histograms. Continuous variables were described using the terms minimum, maximum, mean and standard deviation. The scores found in the cognitive tests were described as percentages of normal and impaired, according to the cutoff points validated for the Brazilian population. Categorical variables were described by percentage and N.

Association analysis was performed between outcome (cognitive results categorized as normal or impaired) and contextual variables (*e.g*. medications, other associated diseases, clinical diagnosis of depression, gender, marital status, BDI score and Epworth score ) using the Fisher’s exact test, except for the MG clinical classification, for which the Pearson’s chi-square test was used. Subsequently, with associations established at p≤0.2, the Poisson regression with robust variance was used. The linearity of the quantitative variables was analyzed and it was found that the assumption of linearity was maintained. In addition, the presence of multicollinearity was assessed using the variance inflation factor (VIF) estimates, noting that the cutoff points are good (close to 1), indicating that the variables are not multicollinear. The statistical significance of the odds ratio indices was assessed using the Wald test. The model's adjustment was assessed using the Hosmer and Lemeshow test. Also, a Pearson correlation was performed between cognitive test scores, contextual variables, and questionnaires based on their gross values, except for the correlation between the MMSE and MoCA tests. For these two, the Spearman’s correlation test was applied. The cognitive scores of patients with and without thymomas were compared, by means of the Student’s t-test, to verify the possible influence of thymomas on cognitive performance. The statistical significance level adopted was p<0.05.

## RESULTS

Eighty-eight patients with MG were initially included; of these, 49 patients were excluded for the following reasons: 39 did not come to the scheduled assessment date, eight did not want to participate in the study, one was hospitalized, and one was under 18 years of age. The final sample of this study consisted of 39 subjects diagnosed with generalized MG. Sociodemographic data are presented in Table 1.

**Table 1.** Descriptive analysis of contextual variables and cognitive scores.

	Minimum	Maximum	Mean	Standard deviation	Normal	Impaired
Age	18	84	51.08	17,133	-	-
Education	0	18	9.31	4,372	-	-
Length of illness	1	38	13.92	9,696	-	-
MMSE	16	30	26.05	3,203	56.4% (22)	43.6% (17)
MoCA	10	29	22.38	4,982	33.3% (13)	66.7% (26)
PVF	0	63	31.00	14,220	79.5% (31)	20.5% (8)
SVF	4	25	16.49	5,201	87.2% (34)	12.8% (5)
A1-A5	15	68	37.38	11,762	41.0% (16)	59.0% (23)
A6	0	15	6.77	4,055	56.4% (22)	43.6% (17)
A7	0	15	6.32	4,101	43.6% (17)	56.4% (22)
MG-QOL	0	46	16.36	15,276	-	-
MGCS	0	34	11.22	8,619	-	-
BDI	0	41	12.49	11,546	-	-
Epworth	0	22	8.21	5,447	61.5% (24)	23.1% (9)
**Medication**	**Use** **% (n)**		**No use** **% (n)**			
Immunomodulators	51.3 (20)		48.7 (19)			
Acetylcholinesterase inhibitors	92.3 (36)		7.7 (3)			
Glucocorticosteroids	59.0 (23)		41.0 (16)			
Antidepressants	38.5 (15)		61.5 (24)			
-	**Yes** **% (n)**		**No** **% (n)**			
Associated diseases	74.4 (29)		25.6 (10)			
Depressionª	17.9 (7)		82.05 (32)			
Thymoma	51.2 (20)		43.5 (17)			

MMSE: Mini-Mental State Examination; MoCA: Montreal Cognitive Assessment; PVF: Phonemic verbal fluency; SVF: semantic verbal fluency; A1-A5: immediate memory; A6: short term memory retention; A7: recent memory; MG-QOL: Myasthenia Gravis - quality of life scale; MGCS: Myasthenia Gravis Composite Scale; BDI: Beck Depression Inventory; ªaccording to medical record.

Regarding the cognitive battery, there was a predominance of impairments, (according to the cutoff points specific for the Brazilian population) in the MoCA screening test (66.7%) and in the subtasks of immediate (59.0%) and recent memory (56.4%) in the RAVLT test. Regarding the self-perception questionnaires, 23.1% of patients presented scores which suggested EDS, based on data from the Epworth questionnaire, and 41.02% presented scores suggestive of depression, according to the BDI (Table 1).

Regarding drug treatment, there was a predominance of anticholinesterase inhibitor (92.3%) use, followed by CG (59.0%). Most patients used more than one type of medication (Table 1). The MGFA clinical classification scores distributed a similar proportion of patients among classes 1 (28.2%; n=11), 2 (33.3%; n=3), and 3 (30.8%; n=12), whereas only a smaller number of patients were assessed as class 4 (7.7%; n=3). Concerning the BDI classification, it was possible to observe 48.7% (19) of subjects without scores of depression, 12.8% (5) with scores of mild to moderate depression, 20.5% (8) with moderate to severe depression, and 7.7% (3) with severe depression.

Correlation analyses were performed between the gross scores of the cognitive tests, clinical variables, and questionnaire scores, as presented in Tables 2 and 3. Positive correlations were found between all cognitive tests and level of education, showing that the higher the education, the higher the test scores. Age correlated only with the RAVLT test, demonstrating that the younger the patient, the better the individuals’ performance on memory tasks. Regarding quality of life, no correlation was found between MG-QOL and cognitive tests. On the other hand, a positive correlation was established between MG-QOL and Myasthenia Gravis Composite Scale (MGCS), showing that patients with a poorer perception of quality of life also had more severe motor impairments.

**Table 2.** Correlations between clinical variables and cognition.

Cognitive Tests	Age		Education		Length of illness	
	p-value	r	p-value	r	p-value	r
MMSE	0.738	-	<0.001	0.602 ^2^	0.728 ^2^	-
MoCA	0.380 ^2^	-	<0.001	0.695 ^2^	0.806 ^2^	-
PVF	0.455 ^2^	-	<0.001	0.623 ^2^	0.773 ^2^	-
SVF	0.336 ^2^	-	<0.001	0.628 ^2^	0.367 ^2^	-
A1-A5	<0.00 1	- 0.591	0.001	0.50 8 ^2^	0.778 ^2^	-
A6	<0.001	-0.663 ^2^	0.097 ^2^	-	0.502 ^2^	-
A7	<0.001	-0.643 ^2^	0.014	0.397 ^2^	0.324 ^2^	-

MMSE: Mini-Mental State Examination; MoCA: Montreal Cognitive Assessment; PVF: phonemic verbal fluency; SVF: semantic verbal fluency; A1-A5: immediate memory; A6: short term retention memory; A7: recent memory; ^1^ Spearman's Correlation; ^2^ Pearson Correlation

**Table 3.** Correlations between questionnaires and cognitive tests.

	MG-QOL		BDI		Epworth	
	p-value	r	p-value	r	p-value	r
Age	0.306 ^2^	-	0.157 ^2^	-	0.328 ^2^	-
Education	0.352 ^2^	-	0.609 ^2^	-	0.313 ^2^	-
Length of illness	0.114 ^2^	-	0.776 ^2^	-	0.967 ^2^	-
MMSE	0.101 ^2^	-	0.001	-0.551 ^2^	0.582 ^2^	-
MoCA	0.628 ^2^	-	0.155 ^2^	-	0.678 ^2^	-
PVF	0.995 ^2^	-	0.253 ^2^	-	0.822 ^2^	-
SVF	0.609 ^2^	-	0.157 ^2^	-	0.571 ^2^	-
A1-A5	0.796 ^2^	-	0.218 ^2^	-	0.675 ^2^	-
A6	0.048	0.777 ^2^	0.617 ^2^	-	0.162 ^2^	-
A7	0.333 ^2^		0.713 ^2^	-	0.111 ^2^	-
MG-QOL	-	-	0.007	0.449 ^2^	0.491 ^2^	-
MGCS	<0.001	0.775 ^2^	0.054 ^2^	-	0.085 ^2^	-
BDI	0.007	0.449 ^2^	-	-	0.089 ^2^	-
Epworth	0.491 ^2^	-	0.089 ^2^	-	-	-

MMSE: Mini-Mental State Examination; MoCA: Montreal Cognitive Assessment ; PVF: phonemic verbal fluency; SVF: semantic verbal fluency; A1-A5: immediate memory; A6: short term memory retention; A7: recent memory; BDI: Beck Depression Inventory; MG-QOL: Myasthenia Gravis - quality of life scale; MGCS: Myasthenia Gravis Composite Scale; ^1^Spearman's correlation; ^2^ Pearson correlation.

The motor scale (MGCS) only correlated with the RAVLT subtask that evaluates short-term memory (A6) (p=<0.001 and R= -0.663); thus, patients with less motor impairment due to the disease performed better on the memory test. In addition, when patients were divided into with and without thymoma, no correlation was found between thymoma and worse performance in cognitive testes.

After the Poisson regression analysis with robust variance, it was found that patients diagnosed with depression had prevalence ratio (PR)=1,887 (CI 1,166‒3,054) for lower MoCA scores, as well as PR=9,533 (CI 1,600‒56,788) for impairment on PVF tasks and PR= 12,426 (IC 2,177‒70,931) for SVF tasks. Participants who used GC and presented BDI scores indicating depression showed PR=11,227 (CI 1.736‒72.604) and PR=0.351 (CI 0.13‒0.904), respectively, representing lower scores on the RAVLT subtask that assesses short-term memory (A6). Therefore, there is a significant PR for the presence of cognitive deficits in patients with depression and who used GC. No association was observed between the MGFA clinical classification scores and cognitive tasks (Tables 4 and 5).

**Table 4.** Association between cognitive tests and contextual variables.

	MMSE	MoCA	PVF	SVF	A1 - A5	A6	A7
Other diseases¹	0.721	0.056 *	0.086 *	1.00	0.264	0.464	0.062
Depression¹	0.300 *	0.073 *	0.002 *	0.032 *	0.678	0.438	1,000
Antidepressants	-	-	0.037	-	-	-	-
Immunomodulators¹	1,000	0.741	0.695	0.661	0.748	0.341	0.743
Inhibitors¹	0.243	0.253	1,000	1,000	0.557	1,000	0.562
Glucocorticosteroids¹	0.325	1,000	0.109 *	0.631	0.509	0.001 *	0.047 *
Sex¹	0.168 *	0.714	1,000	1.00	0.726	0.299	0.504
Marital Status¹	0.106 *	1,000	1,000	0.349	1,000	0.05 *	0.342
BDI¹ classification	0.182 *	1,000	0.207	0.312	0.727	0.041 *	0.484
Epworth¹ classification	0.698	0.681	1,000	0.545	1,000	0.021 *	1,000
MG² classification	0.897	0.898	0.414 *	0.630	0.340	0.406	0.355

^1^Fisher's exact test; ^2^Pearson’s chi-square test; *proven association; MMSE: Mini-Mental State Examination; MoCA: Montreal Cognitive Assessment; PVF: phonemic verbal fluency; SVF: semantic verbal fluency; A1-A5: immediate memory; A6: short term memory retention; A7: recent memory.

**Table 5.** Regression analysis between cognitive tests and contextual variables

	MMSE			MoCA			PVF			SVF			A6			A7º
	p-value	PR	CI	p-value	PR	CI	p-value	PR	CI	p-value	PR	CI	p-value	PR	CI	p-value
Glucocorticosteroids	-	-	-	-	-	-	0.154	-	-	-	-	-	0.011	11.22	1.73‒72.60	0.071
Other diseases	-	-	-	0.163	-	-	-	-	-	-	-	-	-	-	-	0.123
Depression (1)	0.223	-	-	0.010	1.88	1.16‒3.05	0.013	9.53	1.600‒56.78	0.005	12.42	2.17‒70.93	-	-	-	-
Depression (2)	-	-	-	0.009	0.274	0.18‒1.25	0.084	-	-	-	-	-				
Class 2	0.010	0.20	0.06‒0.68	0.793	-	-	0.321	-	-	0.343	-	-	0.225	-	-	-
Class 3	0.025	0.25	0.07‒0.84	0.236	-	-	0.437	-	-	0.338	-	-	0.058	3.72	0.95‒14.46	-
Class 4	0.055	0.23	0.05‒1.02	0.611	-	-	0.275	-	-	0.278	-	-	0.103	-	-	-
Marital status	0.412	-	-	-	-	-	-	-	-	-		-	0.160	-	-	-
BDI	0.174	-	-	-	-	-	-	-	-	-	-	-	0.030	0.35	0.13‒0.90	-
Epworth	-	-	-	-	-	-	-	-	-	-	-	-	0.786	-	-	-
Gender	0.053	3.04	0.98‒9.42	-	-	-	-	-	-	-	-	-	-	-	-	-

Poisson regression with robust variance; ºgross analysis - p=0.057; MMSE: Mini-Mental State Examination; MoCA: Montreal Cognitive Assessment; PVF: phonemic verbal fluency; SVF: semantic verbal fluency; A1-A5: immediate memory; A6: short term memory retention; A7: recent memory; BDI: Beck Depression Inventory; PR: prevalence ratio ; CI: confidence interval; (1) no inclusion of the antidepressant use variable; (2) inclusion of the antidepressant use variable.

## DISCUSSION

To the best of our knowledge, so far, this study has been the first to investigate cognitive performance in patients with MG in the Brazilian population. The results of this study showed worse performance in tasks related to memory in patients with MG. Moreover, this change was associated with depression and the use of GC. These data corroborate the findings of the systematic reviews by Mao et al.^16^ and Paul et al.,^1^ in which the authors point out that, although there are several studies also pointing to a cognitive decline in MG, they did not exclude the possibility that cognitive function may have been affected by other aspects such as sleep apnea, depression and Type 1 drug use. In their review, Paul et al.^1^ already mentioned the adverse effect of high doses of drugs, such as prednisone, as well as depression on the cognitive functioning of patients with MG. In addition, our results show cognitive decline in the same functions highlighted by other studies, such as memory^5^ ^,^ ^6^ ^,^ ^7^ ^,^ ^8^ ^,^ ^9^ ^,^ ^10^ and executive functions.^8^ ^,^ ^10^

There are few studies in the specialized literature that analyzed the interference of MG medication, depression and EDS on cognitive performance. Three studies^9^ ^,^ ^10^ ^,^ ^11^ found a higher incidence of cognitive impairment in patients with depression or scores suggestive of depression in self-perception questionnaires. Three other studies describe an analysis of cognitive performance and the use of MG medication. Bartel and Lotz^29^ published a paper on a possible association between medications and cognitive impairment. In a linear regression analysis, Marra et al.^14^ found that longer treatment time with CG seemed to be correlated with better performance on attentional tasks and long-term verbal memory, contrary to the evidence in our sample. Interestingly, Jordan et al.^15^ found no association at all between the use of acetylcholinesterase inhibitors and performance in cognitive tests.

Additionally, with our sample, we investigated whether the presence of thymomas could influence patients’ cognitive performance. Our data corroborated other studies^14^ ^,^ ^15^ which found that they did not influence cognitive performance.

### Memory decline and glucocorticosteroid use

Regarding the association between impairments in short-term memory retention and the use of GC, several studies^30^ ^,^ ^31^ ^,^ ^32^ ^,^ ^33^ ^,^ ^34^ have been found on other clinical populations (*e.g*. patients with asthma, rheumatoid arthritis, kidney transplants, and non-CNS systemic diseases), in which participants showed significantly worse performance in memory and attention tests when compared to the control group. Also, research^35^ ^,^ ^36^ on healthy volunteers, with no history of systematically prescribed corticosteroid therapy, noted a significant reduction in performance in memory-related tasks after first-time prednisone use.

Nevertheless, it bears stressing that, while there is evidence of cognitive impairment after the use of GC by MG patients, only two studies have investigated this association. Even so, data were not conclusive. Additionally, they investigated only prednisone^14^ and AI.^15^ The pathophysiology of the adverse effects of using synthetic GC is still unclear.^30^ ^,^ ^35^ ^,^ ^37^

Therefore, the risk of memory impairment should be considered before starting treatment with GC and in monitoring patients with MG. Thus, with the other forms of monitoring already routinely performed on MG patients treated with GC, cognitive aspects should also be taken into consideration - such as memory - in addition to psychiatric symptoms such as depression^31^ and the importance of a proactive approach on the part of health professionals who follow patients with MG, as these patients may not have self-perception of cognitive changes. Therefore, they should be investigated regardless of patients’ complaints.

### Losses in cognitive function associated with depression

In our sample, there was a significant prevalence ratio of depression in participants who showed cognitive impairment on screening tests, where verbal fluency and memory are concerned. These results corroborate the data in the literature that patients with depression present cognitive decline, regardless of severity (*i.e.* whether mildly or severely depressed). Studies have shown poorer performance in: memory tests (on tasks such as coding, retrieval, recall, and recognition),^37^ ^,^ ^38^ ^,^ ^39^ ^,^ ^40^ ^,^ ^41^ sustained and/or selective attention, psychomotor deceleration (such as reaction time, information, writing, and drawing tasks),^38^ ^,^ ^39^ ^,^ ^42^ verbal fluency,^37^ ^,^ ^39^ ^,^ ^40^ ^,^ ^43^ and executive function.^38^ ^,^ ^39^ ^,^ ^41^ ^,^ ^42^

The causes of these deficits can be explained by three different theories: 1) the stress hypothesis, which proposes that performance in stress tasks is disproportionately impaired in depressed patients when compared to their performance in automatic ones; 2) the cognitive velocity hypothesis, which states that depression is characterized by cognitive slowness and that this deceleration may be at the root of other cognitive impairments, and 3) the hypothesis of impairment of executive control functions which, in turn, is underlying to the hypothesis of effort.^44^ The present study fortifies theories 1 and 2 of these authors.^44^

### Limitations of the study

The cross-sectional and exploratory design of the study presented limitations, since it did not allow for analysis of the causal factors of the cognitive decline found in the sample. Thus, we identified a need for longitudinal studies that could explain whether cognitive impairment is due to the pathophysiology of the disease or associated with other clinical aspects.

Another limitation of the study is that it was carried out at a reference public hospital in the treatment of MG patients. This may have led more severe patients to our recruitment universe, as they require more specialized care. As such, the representative power of the sample may have been reduced. Moreover, we cannot analyze the correlation between antibodies and cognitive performance, as the test was not available in the public health system

In addition, considering the known differences related to education and socioeconomic levels of other populations, the scarcity of studies on the Brazilian population with MG did not allow for a comparison between the scores found in this sample.

In this sample, participants with MG presented worse performance in tasks of executive function and immediate and recent memory. These are not associated with the time and severity of the disease. However, a significant prevalence ratio was found for poorer memory performance in patients diagnosed with depression and in those using GC. Regarding QoL, only the motor scale showed a positive correlation, suggesting that patients with a poorer perception of quality of life also suffered from more severe motor restrictions.
